# Differential Roles of Two Leptin Gene Paralogues on Food Intake and Hepatic Metabolism Regulation in Mandarin Fish

**DOI:** 10.3389/fendo.2020.00438

**Published:** 2020-08-14

**Authors:** Xiao-Chen Yuan, Xu-Fang Liang, Wen-Jing Cai, Ai-Xuan Li, Dong Huang, Shan He

**Affiliations:** ^1^College of Fisheries, Chinese Perch Research Center, Huazhong Agricultural University, Wuhan, China; ^2^Key Lab of Freshwater Animal Breeding, Ministry of Agriculture and Rural Affair/ Hubei Engineering Technology Research Center for Fish Breeding and Healthy Aquaculture, Wuhan, China

**Keywords:** fish leptin, food intake, appetite, hepatic glycogen, gluconeogenesis, triglyceride

## Abstract

Leptin affects food intake regulation and energy homeostasis in mammals, as opposed to mammals who have a single leptin gene, fish have duplicated leptin gene paralogues. Until now, most functional studies on fish focused on the first reported paralogue without much explanation on specific gene paralogue. This study successfully expressed two homologous recombinant mandarin fish leptin genes (LepA and LepB) for the first time. To explore the differential roles of these two gene paralogues involved in food intake and energy homeostasis, mandarin fish were treated with homologous recombinant LepA and LepB proteins by acute IP administration. The results showed that LepB inhibited the food intake of mandarin fish after acute IP administration through modifying the expressions of hypothalamic orexigenic genes, while LepA had no significant effect on its food intake. In addition, LepB administration decreased the hepatic glycogen level through regulating the gene expressions of glycogen synthase and glycogen phosphorylase in mandarin fish until 4 d, while LepA did not change the hepatic glycogen level as it failed to change the expressions of these regulatory genes. Moreover, LepA and LepB downregulated the expressions of key gluconeogenic genes (phosphofructokinase, phosphoenolpyruvate carboxykinase, and glucose-6-phosphatase), indicating both mandarin fish leptins could regulate the rate of glucose production. However, these two gene paralogues presented secondary effects on lipid metabolism as they only enhanced the triglyceride level by modifying the gene expressions of adipose triglyceride lipase or acetyl CoA carboxylase just for 1 d after IP. Therefore, LepB played an important role in food intake and glucose homeostasis regulation, while LepA showed a limited role in gluconeogenesis and lipid metabolism.

## Introduction

Leptin plays an important role in weight control, lipid metabolism, energy homeostasis, and reproduction in mammals (1, 2). A large amount of studies on leptin have been carried out all over the world (>823,000 published manuscripts; ISI search April 2020). Moreover, leptin has been regarded as a satiety signaling factor in food intake regulation in recent decades (3–5).

As opposed to mammals who have a single leptin gene, fish leptins diverged into duplicated leptin gene paralogues (6–8) after a major genome replication event in the fish lineage about 300 million years ago. The divergent leptin gene paralogues have been cloned in several species of fish, including zebrafish (*Danio rerio*) (7), carp (*Cyprinus carpio*) (9), Atlantic salmon (*Salmo Salar*) (10, 11), medaka (*Oryzias latipes*) (12), and orange-spotted grouper (*Epinephelus coioides*) (13). However, the differential functions of the two leptin gene paralogues were only studied in goldfish (*Carassius auratus*), zebrafish and White-clouds Mountain minnow (*Tanichthys albonubes*), and limited to the effect of energy on the gene expression of divergent leptins (14–16).

The functional studies of leptin have been gradually proceeded since its discovery. Acute and chronic intraperitoneal injection (IP) leptin administrations reduced food intake and body weight in goldfish, which were consistent with the results in mammals (17). However, peripheral treatment with mammalian leptin in immature coho salmon (*Oncorhynchus kisutch*) (18) or green sunfish (*Lepomis cyanellus*) (19) did not change their feeding behavior or energy metabolism. The contradictory results are probably due to the large difference in the amino acid sequences of leptin between fish and mammals. Therefore, it is indispensable and important to use the homologous protein to study the physiological function of leptin on fish.

Mandarin fish (*Siniperca chuatsi* Basilewsky) which is a demersal piscivore, is found only in the freshwaters of China and the River Amur along the Russian borderlands (20). It is one of the chief economic freshwater fishes in China due to its fast growth and delicious flesh (21). In recent years, the aquaculture of mandarin fish has been developed rapidly. The production of this fish overall was 315,906 t in the year 2018 and this amount is only from China. In addition, it has a unique food preference, since it feeds solely on live fry of other fish species and once the fry start feeding in the wild (22). The mandarin fish leptin A (LepA) and leptin B (LepB) genes have been cloned in our previous study (23). In the present study, to explore the differential functions of leptin on food intake and energy metabolism, we treated mandarin fish with the homologous recombination LepA and LepB through intraperitoneal injection.

## Methods and Materials

### Animals and Samples

The experimental mandarin fish (initial body weight: 14.58 ± 0.33 g) was purchased from Wuhan Sihui Fishery (Wuhan, Hubei, China) and stored in a 300-L tank at Huazhong Agricultural University. Animal experiments were approved by the ethical committee of Huazhong Agricultural University. The fish were acclimated to the experimental conditions for 2 weeks. During acclimation, fish were fed to apparent satiation with *Cirrhinus mrigala* twice a day at 9:00 and 17:00, respectively. After the 2-weeks acclimation, six mandarin fishes used for the cloning and recombinant expression of leptin proteins were randomly selected and then anesthetized with MS-222 (200 mg/L). The liver samples were collected and frozen immediately in liquid nitrogen and stored at −80°C until used.

### Construction of Recombinant Plasmids of Leptins

Liver RNA extraction and cDNA transcription were performed with Trizol reagent (Takara, Japan) and PrimeScript™ RT reagent Kit with gDNA Eraser (Takara) according to the manufacturer's protocols. The quantity and quality of RNA obtained were checked by spectrophotometric analysis with the Eppendorf Biophotometer Plus; ratio of absorbance at 260 and 280 nm (*A*_260_/*A*_280_) was used to assess purity of RNA and *A*_260_/*A*_280_ ratios above 2.0 for RNA were controlled.

The full-length of mRNA sequences of the mandarin fish leptins were obtained from our previous study (23). Primers were designed on the complete coding sequences of mature proteins (Table 1) referred to the ClonExpress II One Step Cloning Kit (Vazyme Biotech, Jiangsu, China). Then, using cDNA from the liver of mandarin fish as the template, the products of complete coding sequence of LepA and LepB mature proteins were amplified first. The PCR products were purified from agarose gel and verified by DNA sequencing (Sangon Biotech, Shanghai, China). The purified PCR products of complete coding sequences of LepA and LepB mature proteins were then subcloned into the pET30a vector (Invitrogen, Carlsbad, CA) using ClonExpress™ II One Step Cloning Kit (Vazyme Biotech), respectively. The recombinant LepA-pET30a and LepB-pET30a plasmids were transformed into *E. coli* DH5α using heat shock transformation. Positive transformants were selected, and inserts were sequenced to confirm the proper sequence.

**Table 1:** Primers of recombinant plasmids construction.

**Primer**	**Sequence 5^**′**^-3^**′**^**	**AL (bp)**	**Genbank**
LepA-lFor	GTACCGACGACGACGACAAGGCTCCTCTGCCAGTGGAAGTA	449	KC778775
LepA-sRev	TTATCAGCAAGTCTTAAGATGATCCAGATTTTTC		
LepA-sFor	GCTCCTCTGCCAGTGGAAGTAGTGAAG	449	
LepA-lRev	GACGGAGCTCGAATTCGGATTTATCAGCAAGTCTTA		
LepB-lFor	GTACCGACGACGACGACAAGCTTCCCACGAAGGGAGACTCCATC	437	KP993203
LepB-sRev	TTAGCAGACCTTGAGTTTGTCTTTGTTGAGAAG		
LepB-sFor	CTTCCCACGAAGGGAGACTCCATCAG	437	
LepB-lRev	GACGGAGCTCGAATTCGGATTTAGCAGACCTTGAGTTTGTCTTTG		
pET30a-lFor	ATCCGAATTCGAGCTCCGTCGACAAG	5,963	
pET30a-sRev	CCAGATCTGGGCTGTCCATGTGCT		
pET30a-sFor	GACAAGCTTGCGGCCGCACT	5,863	
pET30a-lRev	CTTGTCGTCGTCGTCGGTACCC		

### Protein Expression and Purification

The recombinant LepA-pET30a and LepB-pET30a plasmids were transformed into the competent BL21 (DE3) *E. coli* strain for protein expression. Transformed cells were grown in 4 L of Luria-Broth (LB) medium containing 30 μg/mL ampicillin at 37°C. When absorbance at 600 nm reached 0.6, IPTG (isopropyl b-D-1-thiogalactopyranoside) was added to a final concentration of 0.5 mM. Cells were cultivated for another 4 h and then they were harvested by centrifugation at 6,000 g for 15 min at 4°C. The cells were resuspended in 100 ml of cell lysis buffer (8 M Urea, 50 mM Tris, 300 mM NaCl, 1 mM DTT, 0.2 % TritonX-100, pH = 8.0), and disrupted by sonication. Inclusion bodies were separated from the cell lysate by centrifugation at 14,000 g for 20 min at 4°C.

A Ni-NTA agarose column was equilibrated with 5 mL binding buffer (8 M Urea, 50 mM Tris, 300 mM NaCl, 1 mM DTT, 0.2 % TritonX-100, pH = 8.0). Then the column was loaded with reaction solution at a rate of 2 mL/min, and then washed with washing buffer (8 M Urea, 50 mM Tris, 300 mM NaCl, 1 mM DTT, 0.2 % TritonX-100, 10 mM/20 mM/50 mM Imidazole, pH = 8.0). The protein was eluted with elution buffer (8 M Urea, 50 mM Tris, 300 mM NaCl, 1 mM DTT, 500 mM Imidazole, pH = 8.0). The eluates were then dialyzed against PBS (pH = 7.5) for 12 h and finally sterile filtered through a 0.45 μm cellulose acetate syringe filter. Eluates were collected and analyzed for SDS-PAGE and Western blotting.

### SDS-PAGE and Western Blotting

The samples of the eluates were subjected to SDS-PAGE on a 12% acrylamide gel to confirm the expected size of the proteins. Protein concentrations of LepA and LepB were determined with a Non-Interference Protein Assay Kit (Sangon Biotech) using the BSA as a standard.

The inclusion body fraction containing the recombinant LepA and LepB was subjected to SDS-PAGE, and the resolved protein bands were electrophoretically transferred to polyvinylidene fluoride (PVDF) membranes (Sangon Biotech). The membranes were blocked with 5% non-fat dry milk in Tris-buffered saline containing 0.5% Triton X-100 at 4°C overnight. The blocked membranes were incubated with 1:1000 dilution of mouse anti-His antibody at 37°C for 0.5 h, and 1:1500 dilution of goat anti-mouse IgG horse radish peroxidase conjugate was added and incubated at 37°C for 0.5 h. Then, the derived proteins were eluted, desalted and freeze-dried. The freeze-dried proteins were dissolved in 0.1% TFA, and then analyzed by 4800 plus MALDI-TOF-TOF Analyzer (AppliedBiosystems, USA). The data obtained from the first and second mass spectrometric analysis were analyzed by GPS Explore v3.6 software (AppliedBiosystems, USA). The protein was then identified by searching in the local database using MASCOT v2.1 (Matrix Science, UK) software.

### Effects of Acute IP LepA and LepB Administration on Food Intake

The effect of homologous recombinant LepA and LepB proteins we purified as mentioned above on the food intake of mandarin fish was studied by acute IP administration. Experimental mandarin fish were obtained from the tanks at the Huazhong Agricultural University we reared before. After a 2-weeks acclimation, 54 mandarin fishes were deprived of food for 24 h before IP and then anesthetized with MS-222 (100 mg/L). Then, each experimental fish was weighed to calculate the injection dose of IP. After IP, fish were randomly distributed into nine groups. Each experimental group was provided with six duplicate tanks (60 L), and only one mandarin fish was reared in each of these six biology repetitions. The nine experimental groups were set as: Four groups injected with 100, 500, 1,000, and 1,500 ng/g BW LepA protein (dissolved in DPBS, named A100, A500, A1000, and A1500 groups, respectively); Four groups injected with 100, 500, 1,000, and 1,500 ng/g BW LepB protein (dissolved in DPBS, named B100, B500, B1000, and B1500 groups, respectively); The other one group injected with vehicle with equal volume named the control group. The injection doses of LepA and LepB proteins in mandarin fish were referred to the dose reported in previous studies (17, 24–27). Before injection, equal and excessive *C. mrigala* (prey fish, 0.16 ± 0.003 g) was put into each white plastic tank. Then, the numbers of prey fish remaining in the tanks were recorded as photographs before and 2 and 4 h after the injection. The sampling time (2 and 4 h after the injection) of LepA and LepB administrations affected the food take of mandarin fish were referred to the sampling time reported in previous studies (24–26). Three legible pictures clearly showed the number of prey fish that were selected in each white plastic tank at each time, and the average number of the prey fish was taken into account. The food intake of the experimental group = (the initial amount of prey fish – the rest amount of prey fish at 2 or 4 h) × the average weight of prey fish / the body weight of the mandarin fish.

### Effects of Acute IP LepA and LepB Administration on Biochemical Indexes

The effects of homologous recombinant protein LepA and LepB we purified as mentioned above on hepatic glycogen, triglyceride (TG), and cholesterol (CHO) of mandarin fish were studied by acute IP administration. After the 2-weeks acclimation, mandarin fish were deprived of food for 24 h before IP and then anesthetized with MS-222 (100 mg/L). Then, each experimental fish was weighed to calculate the injection dose of IP. After IP, fish were randomly distributed into nine groups, each set with triplicate groups in plastic tanks (300 L). The density of each tank is 40 fishes. The nine experimental groups were set as: Four groups injected with 100, 500, 1,000, and 1,500 ng/g BW LepA protein (dissolved in DPBS, named A100, A500, A1000, and A1500 groups, respectively); Four groups injected with 100, 500, 1,000, and 1,500 ng/g BW LepB protein (dissolved in DPBS, named B100, B500, B1000, and B1500 groups, respectively); The other one group injected with vehicle with equal volume was named the control group. Fish were fed with equal and excessive *C. mrigala* (prey fish, 0.16 ± 0.003 g) during the 7-days maintaining period. At 9:00 am of 1, 2, 4, and 7 d after injection, six fishes were randomly taken out from each tank, then anesthetized with MS-222 (200 mg/L). The sampling time (1, 2, 4, and 7 d after injection) of LepA and LepB administrations affected the biochemical indexes of mandarin fish and were referred to the sampling time reported in previous studies (17, 27, 28). The liver of each fish was collected quickly and stored at −20°C until used for the detection of glycogen, triglyceride, and cholesterol.

### Effects of IP LepA and LepB Administration on Gene Expressions Related to Appetite and Energy Homeostasis

The effects of the homologous recombinant protein LepA and LepB, which we purified as mentioned above on orexigenic (Neuropeptide Y, *npy*; Agouti-related protein, *agrp*) and anorexigenic (Cocaine- and amphetamine-regulated transcript protein precursor, *cart*; Proopiomelanocortin, *pomc*) genes of mandarin fish were studied by acute IP administration. After the 2-weeks acclimation, mandarin fish were deprived of food for 24 h before IP and then anesthetized with MS-222 (100 mg/L). Then, each experimental fish was weighed to calculate the injection dose of IP. After IP, fish were randomly distributed into nine groups, each set with triplicate groups in plastic tanks (300 L). The density of each tank is 50 fishes. The nine experimental groups were set as: Four groups injected with 100, 500, 1,000, and 1,500 ng/g BW LepA protein (dissolved in DPBS, named A100, A500, A1000, and A1500 groups, respectively); Four groups injected with 100, 500, 1,000, and 1,500 ng/g BW LepB protein (dissolved in DPBS, named B100, B500, B1000, and B1500 groups, respectively); The other one group injected with vehicle with equal volume was named the control group. At 2 and 4 h after injection, four fishes were randomly taken out from each tank, then anesthetized with MS-222 (200 mg/L). The brain of each fish was collected quickly, frozen immediately in liquid nitrogen, and stored at −80°C until used for appetite gene expression.

Then, the effects of the homologous recombinant protein LepA and LepB we purified as mentioned above on energy homeostasis genes (Glucose metabolism related genes: Glycogen synthase, *gys*; Glycogen phosphorylase, *pygl*; 6-phosphofructokinase 1, *pfkl*; Phosphoenolpyruvate carboxykinase, *pepck*; Glucose-6-phosphatase, *g6pc*; Lipid metabolism related genes: Acetyl CoA carboxylase, *acc*; Adipose triglyceride lipase, *atgl*) of mandarin fish were studied by acute IP administration. After the sample collection for appetite gene expression, the remaining fish in each tank were fed with equal *C. mrigala* (prey fish, 0.16 ± 0.003 g) during the 7-days maintaining period. At 9:00 am of 1, 2, 4, and 7 d after injection, four fishes were randomly taken out from each tank of DPBS, 1,000 ng/g LepA and 1,000 ng/g LepB groups, then anesthetized with MS-222 (200 mg/L). The liver of each fish was collected quickly, frozen immediately in liquid nitrogen, and stored at −80°C until used for energy homeostasis gene expression.

RNA extraction and cDNA transcription were performed with Trizol reagent (Takara, Japan) and a PrimeScript™ RT reagent Kit with gDNA Eraser (Takara) according to the manufacturer's protocols. Real-time PCR assays were carried out on a quantitative thermal cycler (CFX Connect Real-Time System, BIO-RAD, Hercules, CA, USA) using AceQ® qPCR SYBR® Green Master Mix (Vazyme Biotech) with the designed primers (Table 2). A set of six housekeeping genes (*actb, rpl13a, tub*α*1a, b2m, hmbs*, and *sdha*) were selected from the transcriptome assemblies (29) to test their transcription stability for tissue panel. GeNorm software was used to compute the expression stability values (M) for each gene where a lower M value corresponds to more stable gene expression. The PCR parameters were 95°C for 3 min followed by 40 cycles at 95°C for 10 s, annealing temperature for 30 s, and a melt curve step. Primer PCR efficiencies of the genes ranged from 96.7 to 105.0%. Gene expression levels were quantified relative to the expression of housekeeping genes using the optimized comparative Ct (2^−ΔΔ*Ct*^) value method (30).

**Table 2:** Primer sequences for gene expression.

**Gene name**	**Primer**	**Sequence 5^**′**^-3^**′**^**	**AL (bp)**	**AE (%)**	**Genbank**
**Primers of Candidate Reference Genes Sequences for the Quantitative Real-Time PCR**					
*actb*	sc-actb-F	AGAGGGAAATCGTGCGTGAC	193	102.0	FJ436084
	sc-actb-R	ATACCGAGGAAGGAAGGCTG			
*rpl13a*	sc-rpl13a-F	TATCCCCCCACCCTATGACA	100	100.6	MK770673
	sc-rpl13a-R	ACGCCCAAGGAGAGCGAACT			
*tubα1a*	sc-tubα1a-F	TGGCTTGCTGCCTTCTGT	141	99.9	MT548576
	sc-tubα1a-R	GCGGCTGGTAGTTGATGC			
*b2m*	sc-b2m-F	GAGGTTCGTGCTGTTTCTGG	285	103.9	MT548577
	sc-b2m-R	TTTTGTCCGCTCGTGGGT			
*hmbs*	sc-hmbs-F	GGATGGGAATGGCAAGGTTA	271	98.8	MT548578
	sc-hmbs-R	GGGCAGGTCTTTCAGTGAGTG			
*sdha*	sc-sdha-F	GTTTGATGCTTTGGTGGTAGGA	104	102.2	MT548579
	sc-sdha-R	TGGGGAAGAGCTTGGTGATG			
**Primers of Appetite Genes for the Quantitative Real-Time PCR**					
*npy*	rt-npy-F	GTTGAAGGAAAGCACAGACA	191	103.4	EF554594
	rt-npy-R	GCTCATAGAGGTAAAAGGGG			
*agrp*	rt-agrp-F	GAGCCAAGCGAAGACCAGA	151	101.0	MK770670
	rt-agrp-R	GCAGCACGGCAAATGAGAG			
*cart*	rt-cart-F	CTGCTGTCCGTCATTTGTCAC	171	105.0	MT548580
	rt-cart-R	TGGGATGCTTCCTCTTTTCTC			
*pomc*	rt-pomc-F	GTGTCATCCTCGTTACTGC	162	97.7	MT548581
	rt-pomc-R	GCGACGCTCCTATTCAAT			
**Primers of Energy Homeostasis Genes for the Quantitative Real-Time PCR**					
*acc*	rt-acc-F	TATGCCCACTTACCCAAATGC	129	102.0	MT548582
	rt-acc-R	TGCCACCATACCAATCTCGTT			
*atgl*	rt-atgl-F	TCTACCGAGTCTCCAGGGCA	146	101.3	MK625655
	rt-atgl-R	TAGCAGGGGTCTGTCTGTGTG			
*gys*	rt-gys-F	TACACTGCCTGACCAAGACC	121	99.3	MT548583
	rt-gys-R	TAATGTGGCTGGAGACGAAT			
*pygl*	rt-pygl-F	TCTCCCGTGTTCTTTACCC	205	101.9	MT548584
	rt-pygl-R	GCCATTGCTGGATGAGTG			
*pfkl*	rt-pfkl-F	GCTACCATCAGCAACAACG	155	96.7	MT548585
	rt-pfkl-R	GCCACAGAATCCACCCAT			
*pepck*	rt-pepck-F	CGTGCTGGACTGGATGTTC	104	102.8	MT548586
	rt-pepck-R	CCAAGCCCTGGAGGTTCA			
*g6pc*	rt-g6pc-F	GCCTGTGGATGCTAATGGG	194	101.2	MT548587
	rt-g6pc-R	GGAGGTCAAGAAGAGAGTCGTG			

### Statistical Analysis

All data were presented as mean ± S.E.M (standard error of the mean). The normality of data was assessed by using SPSS software with the Shapiro-Wilk test. All data were subjected to one-way analysis of variance (one-way ANOVA) using the SPSS 17.0 software. Differences between the means were tested by Duncan's multiple range test after homogeneity of variances was checked. Statistical significance was determined at the 5% level.

## Results

### Effects of Acute IP LepA and LepB Administration on Food Intake and Appetite

**Figure 1:**
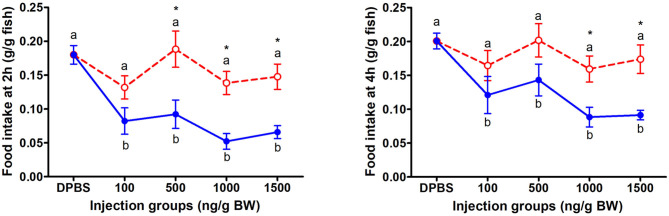
The effects of intraperitoneal administration of LepA (red dotted line) and LepB (blue solid line) on food intake at 2 and 4 h. Data represent means ± S.E.M (*n* = 6). Significance level are marked with different letters (*P* < 0.05), compared with the values from DPBS injected mandarin fish. Mean values with an asterisk (*) above show significant differences between fish groups injected with leptin A and leptin B at the same dose (*P* < 0.05).

The change of food intake in mandarin fish at 2 and 4 h after acute LepA and LepB administration was presented in Figure 1. At 2 and 4 h after IP administration, LepA groups had no significant difference with the vehicle group, while all LepB treated groups had significantly decreased food intake compared with the vehicle group (*P* < 0.05). At 2 h after IP administration under 500, 1,000, and 1,500 ng/g BW doses, the food intake of the mandarin fish was significantly higher under LepA administration than LepB (*P* < 0.05). At 4 h after IP administration under 1,000 and 1,500 ng/g BW doses, the food intake of mandarin fish was significantly higher after LepA administration than LepB (*P* < 0.05).

**Figure 2:**
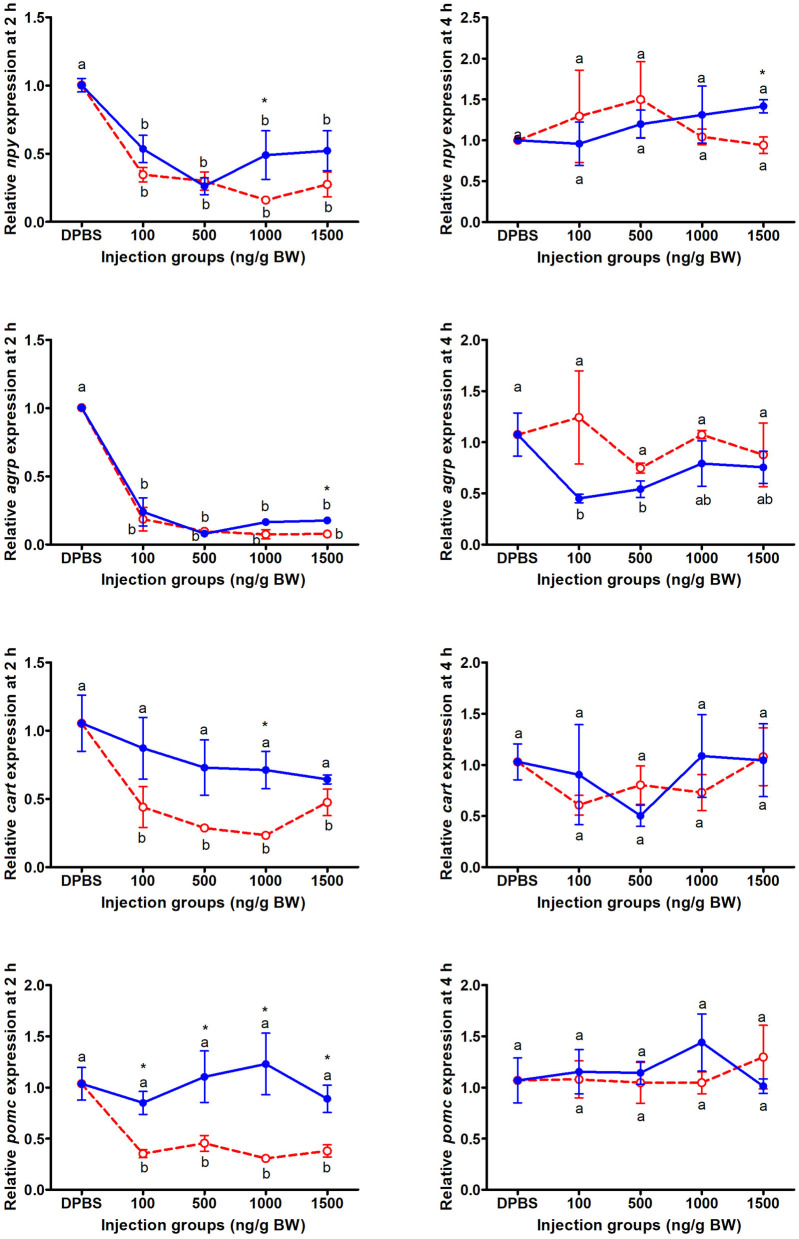
The expressions of appetite genes after acute LepA (red dotted line) and LepB (blue solid line) administration. Data represent means ± S.E.M (*n* = 4). Significance level are marked with different letters (*P* < 0.05), compared with the values from DPBS injected mandarin fish. Mean values with an asterisk (*) above show significant differences between fish groups injected with leptin A and leptin B at the same dose (*P* < 0.05).

At 2 and 4 h after IP LepA and LepB administration, the mRNA abundances of appetite genes in mandarin fish were presented in Figure 2. At 2 h after IP administration, the expressions of orexigenic genes, *npy* and *agrp*, in the brain of mandarin fish treated with LepA and LepB proteins were significantly lower than the fish treated with vehicle (*P* < 0.05). At 2 h after IP administration, the expressions of anorexigenic genes, *cart* and *pomc*, in the brain of mandarin fish treated with LepA protein were significantly lower than the fish treated with the vehicle (*P* < 0.05), while no significantly difference was seen in these two gene expressions between LepB and vehicle administrations. In addition to the gene expression of *agrp* was significantly decreased at 4 h after LepB administration under 100 and 500 ng/g BW doses compared with the control group, no significant differences were observed in the appetite gene expressions among the other LepA and LepB administrations and vehicle administration at 4 h after IP administration.

### Effects of Acute IP LepA and LepB Administration on Glucose Metabolism

**Figure 3:**
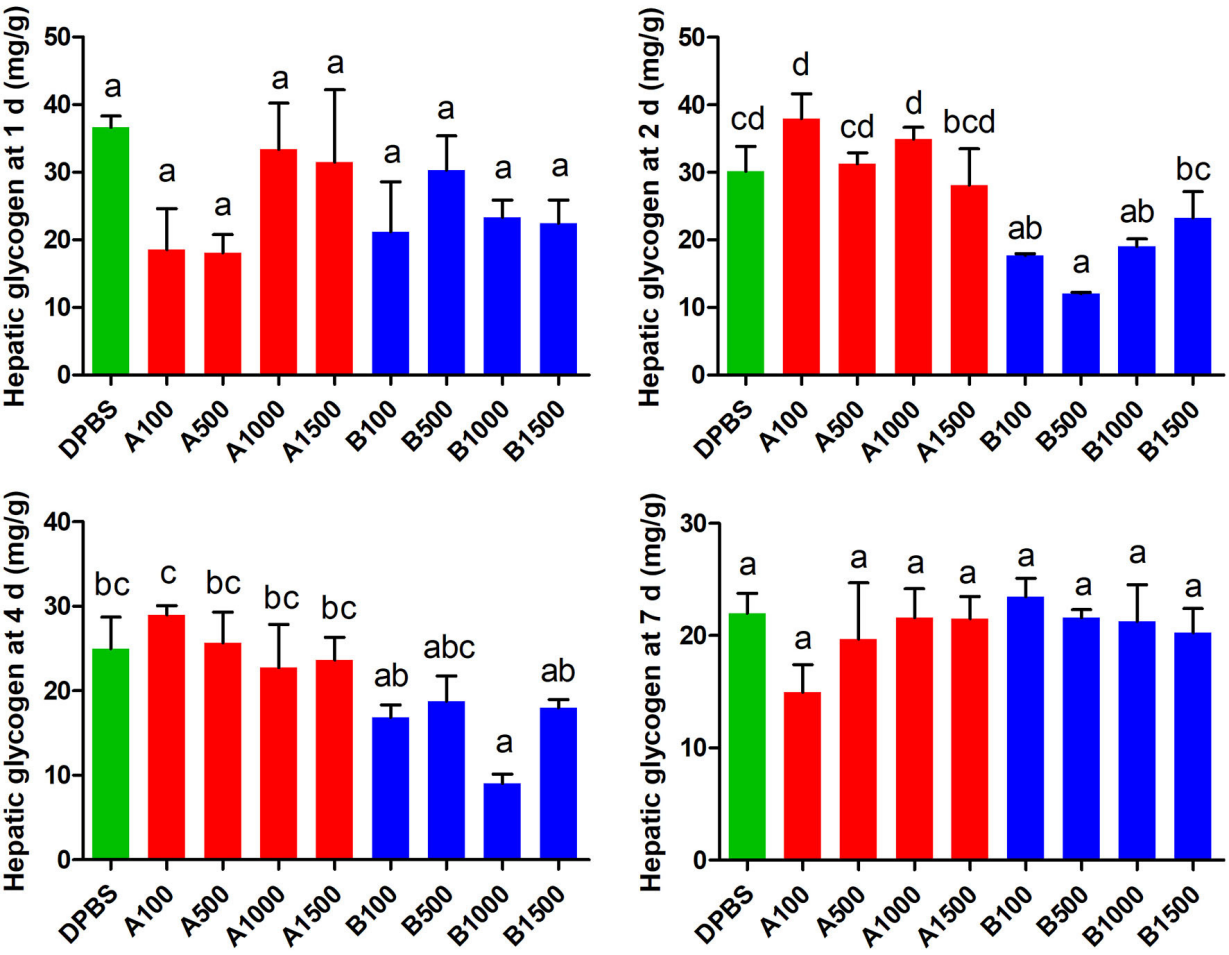
The effects of intraperitoneal injection of LepA and LepB on hepatic glycogen levels at 1, 2, 4, and 7 d. Data represent means ± S.E.M (*n* = 6). The colors of bars were shaded according to intraperitoneal injection of different solvent (green: DPBS, red: LepA, blue: Lep B). Significance level are marked with different letters (*P* < 0.05), compared with the values from DPBS injected mandarin fish.

The effect of acute IP LepA and LepB administration on hepatic glycogen was presented in Figure 3. LepB significantly decreased the hepatic glycogen at 2 d after administration under 100, 500, and 1,000 ng/g BW doses (*P* < 0.05). At 4 d after IP administration, the hepatic glycogen was significantly reduced by the LepB treatment at 1,000 ng/g BW (*P* < 0.05). At 1 and 7 d after IP administration, LepB had not modified the hepatic glycogen. There were no significant differences observed in the hepatic glycogen under acute IP LepA administration.

In order to explore the molecular mechanism of hepatic glycogen changes, we further detected the expressions of key genes related to glucose metabolism by acute IP LepA and LepB administrations at 1,000 ng/g BW treated dose (as shown in Table 3). As expected, the expression of the key enzyme for glycogen synthesis, *gys*, was significantly lower in acute IP LepB administration compared with the vehicle at 1, 2, and 4 d (*P* < 0.05). Correspondingly, the gene expression of glycogen phosphorylase (*pygl*) was significantly enhanced by the IP LepB administration compared with the vehicle at 4 d (*P* < 0.05). No significant difference was seen in *gys* or *pygl* expression under IP LepA administration at different time points compared with the vehicle treatment. Meanwhile, LepA and LepB also shifted the expression of three key genes for gluconeogenesis (*pfkl, pepck*, and *g6pc*). At 1 d after treatments, both LepA and LepB significantly decreased the mRNA abundances of *pepck* and *g6pc* compared with the vehicle (*P* < 0.05). At 2 d after IP administrations, LepA significantly decreased the mRNA abundance of *g6pc*, and LepB significantly reduced the mRNA abundances of *pfkl, pepck*, and *g6pc* compared with the vehicle (*P* < 0.05). The gene expressions of *pfkl* and *pepck* were significantly decreased in both LepA and LepB administration compared with the vehicle at 4 d after treatments (*P* < 0.05). At 7 d after treatments, neither LepA nor LepB could modify the gene expressions of these three gluconeogenic genes.

**Table 3:** The expressions of genes related to energy homeostasis after LepA and LepB administration.

**Time**	**Treatment**	***gys***	***pygl***	***pfkl***	***pepck***	***g6pc***	***acc***	***atgl***
1 d	DPBS	1.02 ± 0.10^b^	0.92 ± 0.20^a^	0.85 ± 0.05^a^	1.02 ± 0.11^b^	1.01 ± 0.09^c^	1.12 ± 0.10^a^	1.07 ± 0.23^b^
	LepA (1,000 ng/g BW)	0.77 ± 0.13^ab^	0.71 ± 0.06^a^	1.31 ± 0.15^a^	0.42 ± 0.15^a^	0.05 ± 0.01^a^	1.58 ± 0.27^ab^	0.28 ± 0.08^a^
	LepB (1,000 ng/g BW)	0.62 ± 0.07^a^	0.94 ± 0.14^a^	1.08 ± 0.22^a^	0.07 ± 0.04^a^	0.43 ± 0.18^b^	2.34 ± 0.33^b^	1.53 ± 0.14^b^
2 d	DPBS	1.23 ± 0.37^b^	1.23 ± 0.49^a^	1.17 ± 0.14^b^	1.17 ± 0.14^b^	1.17 ± 0.08^b^	1.04 ± 0.15	0.90 ± 0.14
	LepA (1,000 ng/g BW)	0.73 ± 0.16^ab^	0.87 ± 0.15^a^	0.99 ± 0.12^ab^	1.12 ± 0.10^b^	0.72 ± 0.20^a^	0.86 ± 0.17	0.89 ± 0.20
	LepB (1,000 ng/g BW)	0.26 ± 0.07^a^	0.93 ± 0.25^a^	0.66 ± 0.07^a^	0.50 ± 0.12^a^	0.71 ± 0.11^a^	1.10 ± 0.45	1.16 ± 0.35
4 d	DPBS	1.02 ± 0.13^b^	0.82 ± 0.22^a^	1.51 ± 0.15^b^	1.55 ± 0.30^b^	0.96 ± 0.33^a^	1.23 ± 0.23	1.32 ± 0.26
	LepA (1,000 ng/g BW)	0.78 ± 0.09^ab^	1.00 ± 0.08^ab^	1.04 ± 0.16^a^	0.84 ± 0.38^a^	0.67 ± 0.09^a^	1.26 ± 0.37	1.28 ± 0.35
	LepB (1,000 ng/g BW)	0.44 ± 0.19^a^	1.47 ± 0.07^b^	0.94 ± 0.04^a^	0.83 ± 0.33^a^	0.52 ± 0.03^a^	1.32 ± 0.18	1.03 ± 0.24
7 d	DPBS	1.07 ± 0.21	1.09 ± 0.26	1.31 ± 0.32	1.08 ± 0.26	0.72 ± 0.10	1.02 ± 0.17	1.48 ± 0.28
	LepA (1,000 ng/g BW)	1.36 ± 0.15	0.96 ± 0.22	1.54 ± 0.36	1.18 ± 0.35	1.30 ± 0.41	1.10 ± 0.29	1.48 ± 0.05
	LepB (1,000 ng/g BW)	0.94 ± 0.16	0.76 ± 0.09	0.94 ± 0.08	1.29 ± 0.36	0.91 ± 0.35	1.11 ± 0.15	1.31 ± 0.30

*Data represent means ± S.E.M (n = 4). Significance level are marked with different letters (P < 0.05), compared with the values from DPBS injected mandarin fish (P < 0.05)*.

### Effects of Acute IP LepA and LepB Administration on Lipid Metabolism

**Figure 4:**
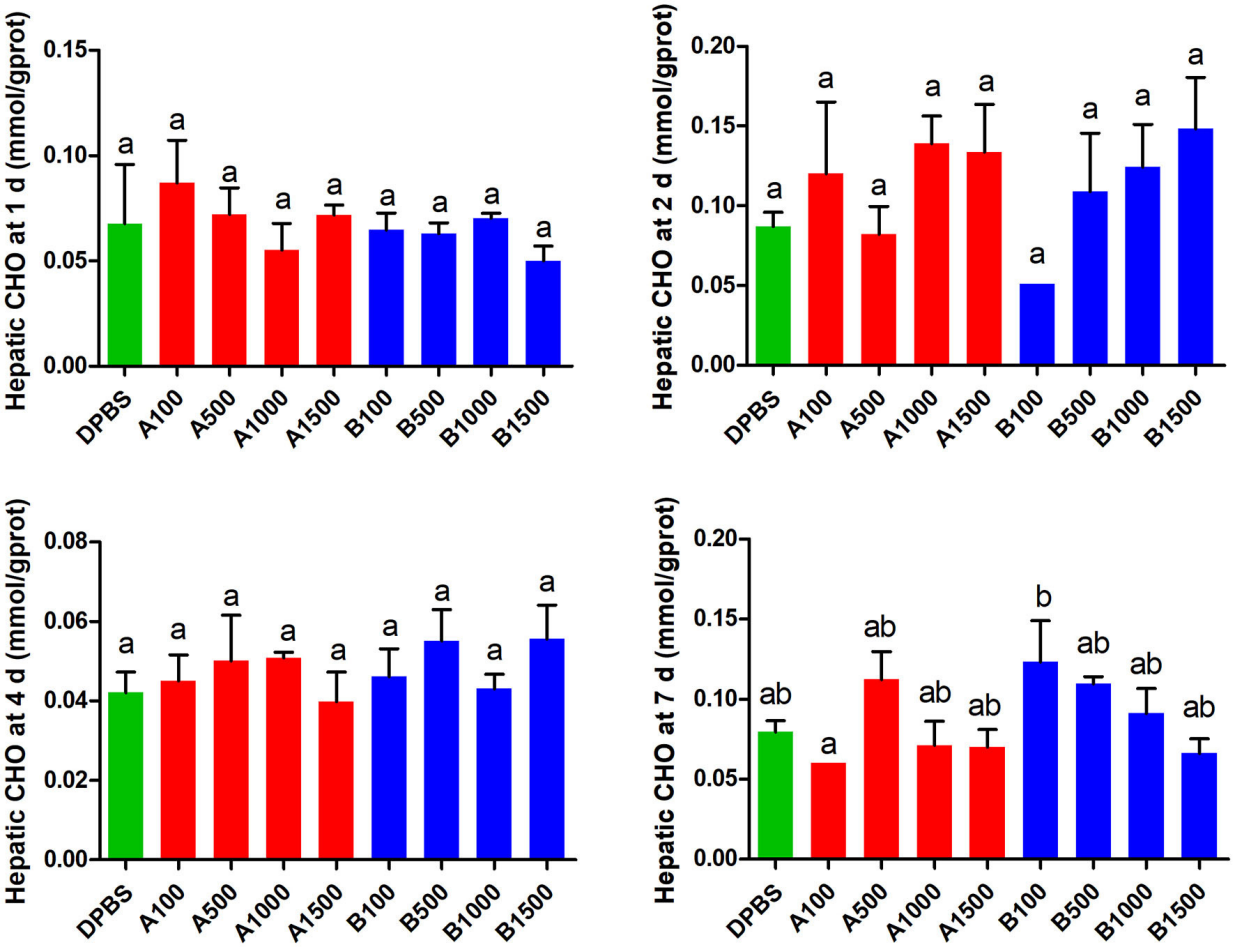
The effects of intraperitoneal injection of LepA and LepB on hepatic CHO levels at 1, 2, 4, and 7 d. Data represent means ± S.E.M (*n* = 6). The colors of bars were shaded according to intraperitoneal injection of different solvent (green: DPBS, red: LepA, blue: Lep B). Significance level are marked with different letters (*P* < 0.05), compared with the values from DPBS injected mandarin fish.

**Figure 5:**
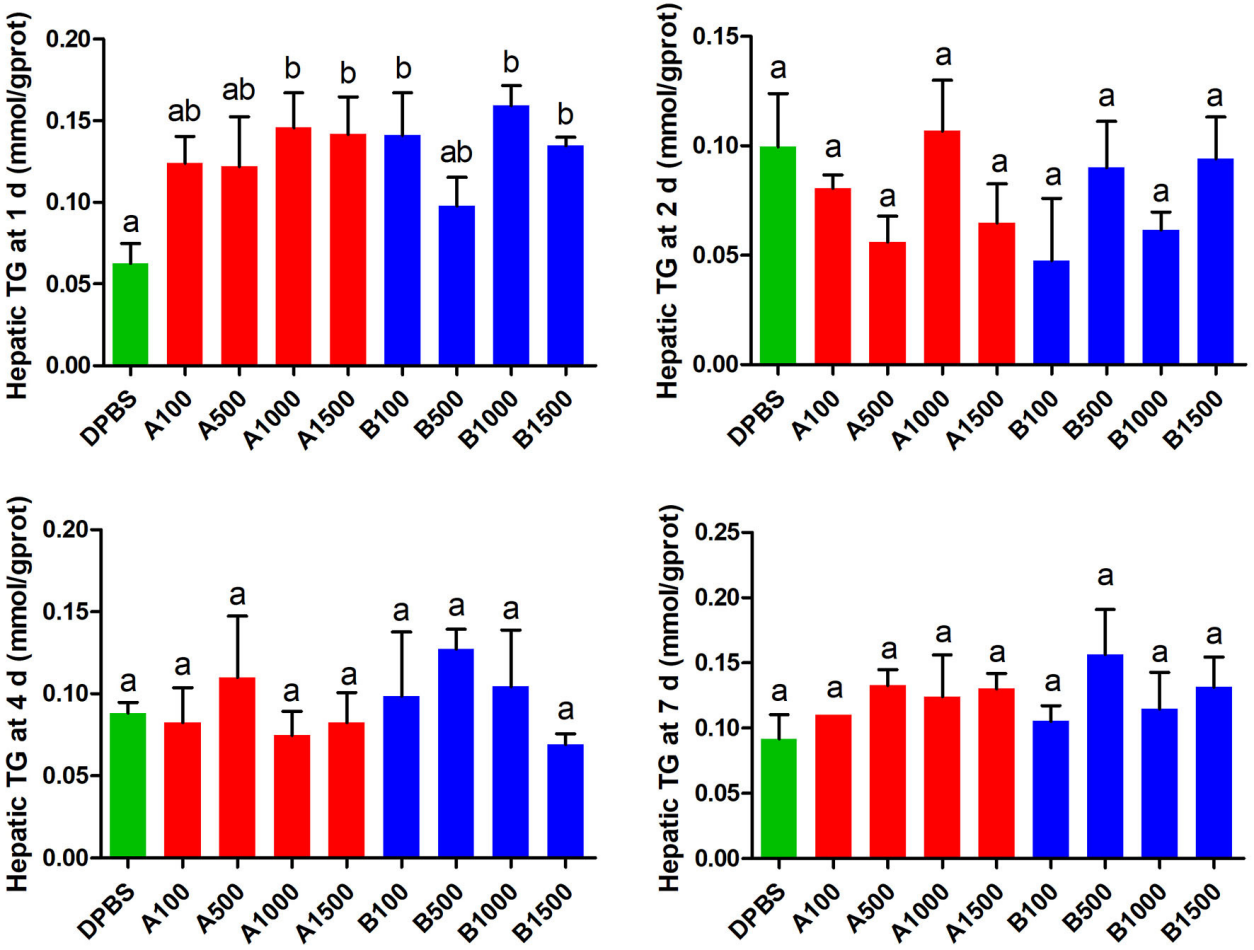
The effects of intraperitoneal injection of leptin A and leptin B on hepatic TG levels at 1, 2, 4, and 7 d. Data represent means ± S.E.M (*n* = 6). The colors of bars were shaded according to intraperitoneal injection of different solvent (green: DPBS, red: LepA, blue: Lep B). Significance level are marked with different letters (*P* < 0.05), compared with the values from DPBS injected mandarin fish.

The effects of acute IP LepA and LepB administration on cholesterol (CHO) and triglyceride (TG) in the liver were presented in Figures 4, 5, respectively. No significant difference was seen in hepatic cholesterol under IP LepA or LepB administration at different time points compared with the vehicle treatment. At 1 d after treatments, LepA significantly enhanced the hepatic triglyceride at 1,000 and 1,500 ng/g BW doses, and LepB significantly increased the hepatic triglyceride at 100, 1,000, and 1,500 ng/g BW doses compared with the vehicle (*P* < 0.05). No significant difference was seen in hepatic triglyceride under acute IP LepA or LepB administration after 1 d compared with the vehicle treatment.

In order to explore the modulator mechanism of hepatic triglyceride changes, we further detected the expressions of key genes related to lipid metabolism by IP LepA and LepB administrations at 1,000 ng/g BW treated doses (Table 3). At 1 d after IP administrations, LepA significantly reduced the mRNA abundance of the key enzyme for triglyceride lipase (*atgl*), and LepB significantly increased the mRNA abundance of the key enzyme for triglyceride synthesis (*acc*) compared with the vehicle group at the same time (*P* < 0.05). But in addition to that, no significant differences were seen in gene expressions of *acc* and *atgl* under acute IP LepA or LepB administration after 1 d compared with the vehicle treatment.

## Discussion

Leptin is involved in energy homeostasis and growth regulation as a satiety signal in mammals (31, 32). However, the relationship between energy state and leptin has not been fully elucidated in fish (14). As opposed to mammals who have a single leptin gene, fish have duplicated leptin gene paralogues. Until now, almost functional studies on fish focused on the first reported paralogue without much explanation on specific gene paralogue (15, 33). Therefore, in order to explore the origin and physiological functions of tetrapod leptins, we successfully expressed leptin A and leptin B in fish for the first time based on our previous identification of the coding sequence of two mandarin fish leptin gene paralogues (23). Then, we further studied the functional differentiation of these two proteins in mandarin fish.

In the present study, LepB of mandarin fish presented an obvious effect of inhibiting food intake after acute IP administration through regulating the expressions of hypothalamic appetite genes. However, LepA had no significant effect on the food intake of mandarin fish. The effect of LepB on food intake in mandarin fish was consistent with that in mammals (34, 35). Leptin presented an inhibitory effect on food intake in most fish. Goldfish injected with recombinant mouse leptin (100 ng/g BW) showed a significant inhibitory effect on food intake within 1 h (24). Study on leptin in grass carp showed that the homologous recombinant protein significantly inhibited food intake and downregulated the expression of orexigenic gene, *npy*, after a short-term IP treatment (2 h) (26). Murashita et al. (25) found that rainbow trout (*Oncorhynchus mykiss*) injected with homologous recombinant leptin intraperitoneally presented inhibited food intake, meanwhile the reduced *npy* and increased *pomc* expression within 2 h, which was in parallel with the function of leptin in mammals on appetite regulation.

NPY and AgRP have been recognized as two of the most effective orexigenic proteins in mammals and fish (36–38). POMC/CART neuron in the hypothalamus of mammals and fish are involved in the appetite suppression mediated by leptin (39–41). In this study, LepB suppressed the appetite of mandarin fish by inhibiting the expression levels of *npy* and *agrp* within 2 h after IP, and it further took effect until 4 h by decreasing the expression levels of *agrp*, which was in line with the decreased food intake induced by acute IP of LepB. In mammals, leptin regulated the food intake through NPY/AgRP and POMC/CART neurons in the arcuate nucleus of hypothalamus (39). However, it seems that LepB suppressed food intake only through the NPY/AgRP neuron in mandarin fish. Interestingly, mandarin fish LepA not only increased expression levels of *npy* and *agrp*, but also enhanced *cart* and *pomc* expressions at 2 h after IP without modifying the food intake of this fish. This result suggested that LepA might stimulate both NPY/AgRP and CART/POMC neurons within 2 h after IP, so that it failed to inhibit the food intake of mandarin fish. The exact physiological significance of this seemingly “ineffective” cycle needs to be further explored. In fact, the differential function of leptin has been preliminarily discussed in zebrafish. Gorissen et al. (7) reported that the mRNA abundance of leptin-b significantly decreased, while the leptin-a expression significantly increased in the liver of zebrafish after a week of fasting. Moreover, the more effective regulation of leptin B on food intake and appetite than leptin A in mandarin fish were similar to the results of our previous study, which claimed that leptin B might be more sensitive to the central nervous system (CNS) control of energy homeostasis than leptin A through a postprandial and short-term fasting treatment (23).

In addition to CNS, leptin also directly acts on the peripheral tissues of mammals, such as promoting glucose production in liver, regulating the efficiency of gluconeogenesis, or stimulating the generation of hepatic glycogen (2, 42). However, different responses in hepatic glycogen to leptin treatment were presented between fish and mammals. The hepatic glycogen of rainbow trout was decreased with the increasing intraventricular injection dose of leptin (43). IP of human or homologous leptin reduced the hepatic glycogen in tilapia (*Oreochromis niloticus*) (28). In this study, the hepatic glycogen of mandarin fish also decreased at 2 and 4 d after IP of LepB in appropriate doses. The difference in response to leptin in hepatic glycogen between fish and mammals prompted us to further detect the change in mRNA abundance of the key genes which regulated the rate of hepatic glycogen production. As expected, LepB administration modified the hepatic glycogen level through regulating the expressions of *gys* and *pygl* in mandarin fish within 4 d after IP. The delayed response might be secondary to a depressed effect of LepB administration on glucose production, while inherent mechanism remains to be further explored. LepA treatment did not affect hepatic glycogen level due to the unchanged expression of these regulatory genes. In mammals, leptin was claimed to affect food intake regulation and eventually glucose metabolism (44). Therefore, the reduced hepatic glycogen under LepB administration might be attributed to its induced decrease in food intake of mandarin fish.

In addition, although recent studies suggested that fish, especially carnivorous fish, lacked the ability to regulate gluconeogenesis (45–48), the expression of key genes involved in gluconeogenesis in mandarin fish after IP of leptins were decreased as in mammals. In rat, leptin was reported to reduce hepatic glucose production by decreasing the synthesis of the key enzyme of gluconeogenesis (49). In this study, LepA and LepB downregulated the expressions of key gluconeogenic genes (*pfkl, pepck*, and *g6pc*), this indicated both mandarin fish leptins could regulate the rate of glucose production.

In recent study on zebrafish, the role for leptin in the regulation of glucose homeostasis was reported to be conserved across vertebrates, whereas its role as an adipostatic factor is likely to be a secondary role acquired during the evolution of mammals (15). In mammals, leptin inhibits fat deposition in liver, mainly by promoting the hydrolysis of fat to increase energy consumption (50, 51). In this study, except the hepatic triglyceride of mandarin fish after 1 d IP of LepA and LepB at partial doses, no more impacts of leptins on cholesterol and triglyceride were observed at other time points, which was in parallel with the changes in expression levels of key genes involved in triglyceride synthesis and hydrolysis. Both LepA and LepB enhanced the triglyceride level at 1 d after IP, paralleling with the decreased expression of *atgl* induced by LepA while the increased expression of *acc* induced by LepB. This result was consistent with the study of Baltzegar et al. (28) which observed a significant increase in hepatic triglyceride within a short period (6 h) after injected tilapia with human leptin with no significant effect after 24 h. Moreover, the discrepancy of hepatic triglyceride in fish and mammals in response to leptin administration might be due to the different secretory organ of leptin. Unlike in mammals, in which the adipose tissue is the primary site for leptin synthesis, in fish leptin it was reported to be mainly expressed in the liver (7, 9).

In summary, LepB of mandarin fish presented an obvious effect of inhibiting food intake after acute IP administration through modifying the expressions of hypothalamic orexigenic genes, while LepA had no significant effect on the food intake of mandarin fish. In addition, acute LepB administration modified the hepatic glycogen level through regulating the expressions of *gys* and *pygl* in mandarin fish until 4 d, while the LepA did not affect the hepatic glycogen level, probably because it failed to change the expression of these regulatory genes. Moreover, LepA and LepB downregulated the expressions of key gluconeogenic genes (*pfkl, pepck*, and *g6pc*), which suggested both mandarin fish leptins had the ability to regulate the rate of glucose production. However, leptins presented a limited effect on lipid metabolism as they only enhanced the triglyceride level by modifying the expression of *atgl* or *acc* just for 1 d after IP. Therefore, LepB played an important role in food intake regulation and glucose metabolism, while LepA played a limited role in gluconeogenesis and lipid metabolism.

## Data Availability Statement

All datasets generated for this study are included in the article/supplementary material.

## Ethics Statement

The animal study was reviewed and approved by the ethical committee of Huazhong Agricultural University.

## Author Contributions

X-CY conceived the study, participated in the design of the study, carried out the laboratory work, participated in data analysis, and wrote the first draft of the manuscript. X-FL designed the study and contributed to the final version. W-JC and A-XL carried out the laboratory work and assisted with data interpretation. DH collected the source animals and assisted with establishing the experimental treatments. SH participated in data analysis. All authors have read and approved the manuscript.

## Conflict of Interest

The authors declare that the research was conducted in the absence of any commercial or financial relationships that could be construed as a potential conflict of interest.

## Abbreviations

*acc*: Acetyl CoA carboxylase
*actb*: Actin beta
*atgl*: Adipose triglyceride lipase
*agrp*: Agouti-related protein
*b2m*: Beta-2-microglobulin
*cart*: Cocaine- and amphetamine-regulated transcript protein precursor
*g6pc*: Glucose-6-phosphatase
*gys*: Glycogen synthase
*pygl*: Glycogen phosphorylase
*hmbs*: Hydroxymethylbilane synthase
*npy*: Neuropeptide Y
*pfkl*: 6-phosphofructokinase 1
*pepck*: Phosphoenolpyruvate carboxykinase
*pomc*: Proopiomelanocortin
*rpl13a*: Large subunit ribosomal protein L13Ae
*sdha*: Succinate dehydrogenase complex flavoprotein subunit A
*tub*α*1a*: Tubulin alpha-1A chain.
